# Genome-Wide Identification of a Novel Eight-lncRNA Signature to Improve Prognostic Prediction in Head and Neck Squamous Cell Carcinoma

**DOI:** 10.3389/fonc.2019.00898

**Published:** 2019-09-18

**Authors:** Bowen Yang, Jiming Shen, Lu Xu, Ying Chen, Xiaofang Che, Xiujuan Qu, Yunpeng Liu, Yuee Teng, Zhi Li

**Affiliations:** ^1^Department of Medical Oncology, First Hospital of China Medical University, Shenyang, China; ^2^Key Laboratory of Anticancer Drugs and Biotherapy of Liaoning Province, First Hospital of China Medical University, Shenyang, China

**Keywords:** head and neck squamous cell carcinoma (HNSCC), long non-coding RNA (lncRNA), prognostic signature, weighted co-expression network construction (WGCNA), gene set enrichment analysis (GSEA)

## Abstract

**Objectives:** LncRNAs are essential survival prognostic indicators with important biological functions in tumorigenesis and tumor progression. This study aimed to establish a long non-coding RNA (lncRNA) signature that can effectively predict the prognosis of patients with head and neck squamous cell carcinoma (HNSCC) and explore the potential functions of these lncRNAs.

**Materials and Methods:** We re-annotated RNA sequencing and obtained exhaustive RNA-seq data of 269 patients with comprehensive clinical information from the GEO database. Then an 8-lncRNA signature capable of predicting the survival prognosis of HNSCC patients and a nomogram containing this signature were established. Weighted Co-expression Network Construction (WGCNA), Gene Set Enrichment Analysis (GSEA), and Gene Ontology (GO) enrichment were then applied to predict the possible biological functions of the signature and each individual lncRNA.

**Results:** Eight lncRNAs associated with survival in HNSCC patients, including AC010624.1, AC130456.4, LINC00608, LINC01300, MIR99AHG, AC008655.1, AC055758.2, and AC118553.1, were obtained by univariate regression, cox LASSO regression, and multivariate regression. Functionally, patients with high signature scores had abnormal immune functions via GSEA. AC010624.1 and AC130456.4 may participate in epidermal cell differentiation and skin development, and MIR99AHG in the formation of cellular structures. Other lncRNAs in the signature may also participate in important biological processes.

**Conclusions:** Therefore, we established an 8-lncRNA signature that can effectively guide clinical prediction of the prognosis of patients with HNSCC, and individuals with high signature scores may have abnormal immune function.

## Introduction

Head and neck squamous carcinoma (HNSCC), the most common and malignant carcinoma affecting the head and neck region, is also the sixth common cancer worldwide (1, 2). In the past few decades, multidisciplinary therapy based on various combinations of surgery, chemotherapy, and radiotherapy has been applied for the management of HNSCC. In addition to the approved immune checkpoint inhibitors such as the second-line treatment (2016), no further progress has been made, resulting in ~50% of HNSCC patients still dying from the disease (3–5). Meanwhile, the prognostic model currently used for HNSCC patients is based on clinicopathological parameters (CPPs), but many cases with the same clinical stage show different outcomes (5, 6). Prognostic signatures based on mRNAs could not be applied clinically (7, 8). Therefore, for HNSCC patients, an efficient prognostic model that can predict the survival prognosis of patients is urgently required.

Long non-coding RNAs (lncRNAs) are non-coding RNAs with more than 200 nucleotides in length. LncRNAs are important biomarkers for the diagnosis and prognosis of tumors because of higher tissue specificity and increased ease of detection compared with mRNAs (9–12). Accumulating evidence indicates lncRNAs play vital roles in the progression and tumorigenesis of tumors, including HNSCC (12–16). For example, LncRNA MIR31HG promotes cell proliferation and tumorigenesis by targeting HIF1A and P21. NEAT1 promotes laryngeal squamous cell cancer by regulating the miR-107/CDK6 pathway. Overexpression of lncRNA H19 promotes the occurrence of HNSCC (17–19). With the development of the high-throughput sequencing technology, more and more lncRNAs have been discovered, and lncRNA signatures associated with HNSCC prognosis have been established, but the functions of lncRNAs in most signatures remain unknown (20–23). Therefore, establishing an integrated lncRNA model associated with prognosis in HNSCC and predicting the functions of respective lncRNAs is of high importance for both patients and clinicians.

To identify lncRNAs associated with prognosis in HNSCC and guide clinical application, we integrated RNA-seq and clinical survival information of 269 patients from the GEO dataset and established a nomogram containing an 8-lncRNA signature. Functional enrichment was performed to predict the potential functions of the hub lncRNAs.

## Materials and Methods

### Patient Information Collection and Study Design

All HNSCC patients were collected from GSE65858 public database (https://www.ncbi.nlm.nih.gov/geo/query/acc.cgi?acc=GSE65858). Here are two criteria used to select desired samples: (1) patients with mRNA expression data and clinical data were selected; (2) survival time of patients was more than 10 days. The platform of mRNA expression analysis of GSE65858 was Illumina HumanHT-12 V4.0 expression beadchip (GPL10558). All selected expression datasets were log2-transformed, then standardized.

### Construction of lncRNA Expression Through Re-annotation

The Illumina probe sequences were obtained from the annotation file GPL10558 and uniquely mapped to the human genome (hg38) by NCBI blast without mismatch. Specific probes of lncRNAs were obtained by matching the chromosomal position of probes to the chromosomal position of lncRNA genes based on annotations from GENCODE (Release 29). For the case where different probes correspond to the same gene, the expression of the gene is taken as the median. LncRNAs were selected based on the following criteria according to the expression values and the calculated median and standard deviation (SD). First, lncRNAs with non-zero values in more than 75% of the cases were included. Second, the median and SD of the lncRNA was required to be larger than 1.

### Cox Survival Analysis and Least Absolute Shrinkage and Selection Operator (LASSO) Regression With 10-fold Cross-Validation

The prognostic value of each lncRNA was firstly calculated in the univariate Cox analysis using R/survival package, and the lncRNAs with *P* < 0.01 were selected as seed lncRNAs for Cox LASSO regression with 10-fold cross-validation (CV). To identify the prognostic value of the lncRNAs, multivariate Cox regression was further performed using R/survival package based on each “significant” lncRNA disclosed in the above steps. A lncRNA with *P* < 0.05 was defined as significant. The corresponding hazard ratio (HR), 95% confidence interval (CI), and *P*-value were collected.

### Development of an Individualized Prediction Model

The OS and Hazard ratios (HRs) were calculated by Kaplan–Meier algorithm and univariate Cox regression analysis, respectively. The log-rank method tested the differences between the survival curves. We used the following formula to construct a prognostic risk score model: *risk score* = *expGene*1 × β*Gene*1+ *expGene*2 × β*Gene*2 + exp *Genen* × β*Genen* (exp, prognostic gene expression level; β, multivariate Cox regression model regression coefficients). The gene signature score as a predictor for HNSCC patients was analyzed in the model. We find out the significant variables through the univariate Cox regression analysis. Candidate variables with a *P*-value < 0.2 on univariate analysis were included in multivariable model. Finally, multivariable Cox regression model began with the clinical candidate predictors as follows: T stage, M stage, and Score. The nomogram model was built by the coefficients of the multivariable Cox regression model.

### Clinical Use

The R-script stdca was (https://www.rdocumentation.org/packages/DecisionCurve/versions/1.4) used to do decision curve analysis (DCA) which can assess the clinical net benefit of different probability thresholds.

### Gene Set Enrichment Analysis (GSEA)

The expression profile data were ranked according to the signature score, and the data were divided into high-risk group and low-risk group by the mean score. Then, we downloaded the h.all.v6.2.symbols.gmt (24) from the GSEA website, and analyzed our data using GSEA version 3.0.

### Weighted Gene Co-expression Network Analysis

The “WGCNA” package in R was applied to performed co-expression network using the expression values of mRNA and lncRNAs screened above. Briefly, we constructed the weighted adjacency matrix using a power function based on a soft-thresholding parameter β. After that, the adjacency was transformed into topological overlap matrix (TOM), and average linkage hierarchical clustering was performed according to the TOM-based dissimilarity measure. In this study, we chose a minimum size (gene group) of 30 for the genes dendrogram and a cut-line (0.25) for module dendrogram and merged some modules.

### Hub LncRNA and Module Function Annotation

To predict the molecular of each candidate lncRNA, lncRNA-related mRNAs were filtered out by WGCNA. The network visualization was performed by Cystoscope software version 3.5.1 (https://cytoscape.org/). An appropriate cutoff of *p*-values < 0.05 and FDR < 0.05 was used. The statistics and data visualization were performed by ClusterProfiler Package in RStudio.

### Validation of MIR99AHG

We compared the expression of MIR99AHG and the relationship with overall survival in HNSCC and normal tissues with the available data from the gene expression profiling interactive analysis (GEPIA). GEPIA is an online tool that provides expression analysis functions for TCGA and GTEx data.

## Results

### Patient Characteristics

**Figure 1:**
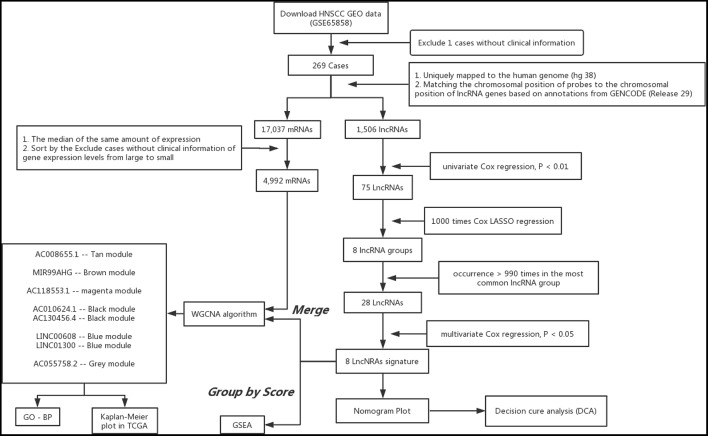
Analysis of flowchart illustrates the exploration procedure for the HNSC prognostic lncRNAs and the related mechanisms.

The analysis procedure of the current study is shown in Figure 1. The basic characteristics of the patients are listed in Table S1. A total of 269 patients with HNSCC were retrieved from the GSE658585 dataset for further analysis. The male to female ratio was 4.72:1, and average age was 60.14 years. There were T3-4 (72.8%) and M0 (97.4%) cases; median survival time was 28 months, and 72.9% of all individuals had no HPV infection.

### Identification of Eight lncRNAs for Predicting HNSCC Patient Survival

**Figure 2:**
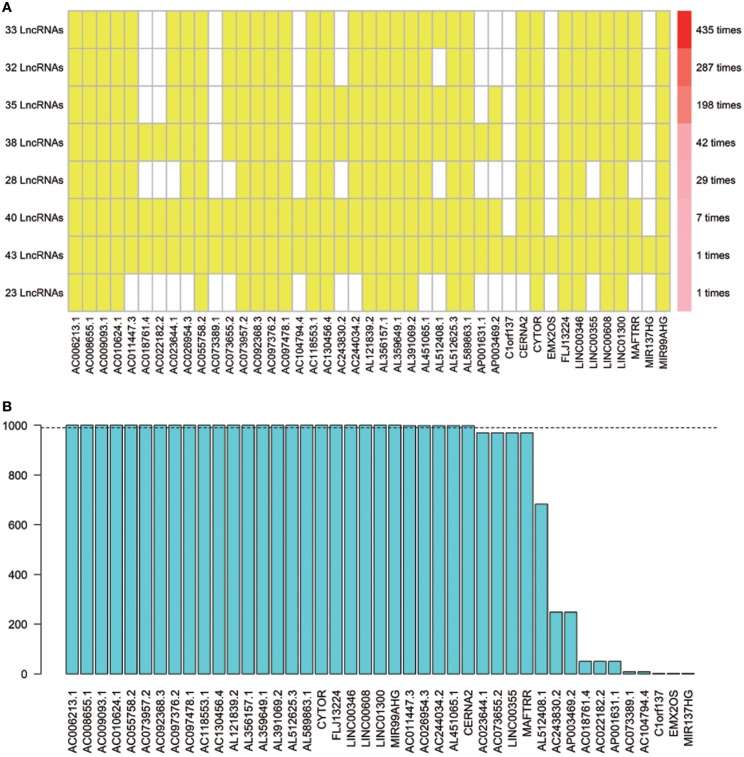
The seed lncRNAs were extracted by 1,000 times Cox LASSO regression. **(A)** Highly consistency was demonstrated in the lncRNAs among the 8 extracted lncRNA sets. The left ordinate indicates the seed lncRNA set and the number of seeds lncRNAs found by every single iteration of LASSO. The right ordinate is the frequency of the seed lncRNA set disclosed through the 1,000 times Cox LASSO regression. The horizontal ordinate is the lncRNA name. The yellow block represents the occurrence of the particular lncRNA in the specific lncRNA set; **(B)** Totally 28 seed lncRNAs with >990 occurrences in the most common lncRNA set were filtered out for further analysis. The blue column indicates the frequency of each lncRNA occurs in the most common lncRNA set.

Using array re-annotation analysis, 1,506 lncRNAs were identified for prognostic significance in univariate Cox survival analysis, and 95 with *P* < 0.01 were filtered out and applied to 1,000 times Cox Lasso regression with 10-fold CV. A total of 8 lncRNA groups were disclosed, and high consistency among the lncRNA sets was demonstrated (Figure 2A). In the most common lncRNA set, 28 lncRNAs were uncovered to show >990 occurrences and extracted for further analysis (Figure 2B). Multivariate Cox analysis based on the 28 lncRNAs finally identified 8 lncRNAs, including AC008655.1, AC010624.1, AC055758.2, AC118553.1, AC130456.4, LINC00608, LINC01300, and MIR99AHG. The detailed information and the survival significance of the 8 lncRNAs are shown in Tables 1, 2.

**Table 1:** Descriptions of the eight lncRNAs.

**Hg38_name**	**Ensembl_ID**	**Havana_gene**	**Gene_type**	**Chr**	**Start**	**End**	**Strand**
MIR99AHG	ENSG00000215386.12	OTTHUMG00000074377.5	lincRNA	21	15928296	16645065	+
AC008655.1	ENSG00000267815.1	OTTHUMG00000183045.1	Antisense	19	50310022	50310539	–
AC010624.1	ENSG00000204666.3	OTTHUMG00000165525.1	Sense_overlapping	19	50050589	50066793	+
AC055758.2	ENSG00000244358.1	OTTHUMG00000159391.1	lincRNA	3	145939912	145961536	+
AC130456.4	ENSG00000260681.1	OTTHUMG00000177223.1	Antisense	16	19,410,729	19,411,662	–
AC118553.1	ENSG00000228084.1	OTTHUMG00000010804.1	Antisense	1	99968383	99969864	–
LINC00608	ENSG00000236445.4	OTTHUMG00000154665.5	Antisense	2	218975393	218989940	+
LINC01300	ENSG00000253595.5	OTTHUMG00000164482.1	Antisense	8	141340549	141344621	+

**Table 2:** The correlations of the lncRNAs with patients' overall survival in HNSCC based on GSE65858 dataset using uni- and multi-variate Cox analysis.

**Gene**	**Univariate cox**			**Multivariate cox**		
	**HR**	**95%CI**	***P*-value**	**HR**	**95%CI**	***P*-value**
AC006213.1	0.02	0–0.25	0.002	0.14	0.01–2.15	0.16
AC008655.1	0.33	0.18–0.63	0.001	0.47	0.23–0.94	0.033^*^
AC009093.1	26.05	2.81–241.84	0.004	9.1	0.45–182.4	0.149
AC010624.1	0.01	0–0.23	0.003	0	0–0.1	0.001^**^
AC011447.3	0.53	0.24–1.17	0.117	0.45	0.16–1.26	0.129
AC026954.3	1.55	0.98–2.47	0.063	1.32	0.73–2.41	0.363
AC055758.2	0.04	0–0.74	0.031	0.02	0–0.67	0.029^*^
AC073957.2	14.28	1.07–190.83	0.044	9.94	0.54–184.8	0.123
AC092368.3	5.29	1.93–14.52	0.001	3.2	0.82–12.55	0.095
AC097376.2	0.04	0–0.46	0.01	0.36	0.02–6.28	0.482
AC097478.1	0.71	0.51–1	0.049	0.89	0.57–1.37	0.589
AC118553.1	0.02	0–0.22	0.001	0.03	0–0.4	0.008^**^
AC130456.4	5.02	1.3–19.41	0.019	22.51	4.02–126.25	0^***^
AC244034.2	0.17	0.04–0.65	0.01	0.99	0.19–4.99	0.986
AL121839.2	5.2	0.68–39.61	0.111	10.86	0.92–127.89	0.058
AL356157.1	12.25	2.18–68.67	0.004	2.51	0.2–32.17	0.479
AL359649.1	30.21	2.05–445.86	0.013	7.09	0.19–260.04	0.286
AL391069.2	0.03	0–0.36	0.005	0.07	0–1.06	0.055
AL451065.1	10.35	1.03–103.75	0.047	2.74	0.12–62.27	0.527
AL512625.3	11.94	1.26–113.15	0.031	8.72	0.57–133.69	0.12
AL589863.1	0.01	0–0.25	0.005	0.09	0–2.54	0.16
CERNA2	0.54	0.33–0.88	0.014	0.7	0.36–1.38	0.305
CYTOR	49.02	4.42–543.27	0.002	1.05	0.06–19.48	0.972
FLJ13224	0.01	0–0.32	0.008	0.03	0–1.13	0.058
LINC00346	21.89	2.24–213.51	0.008	8.91	0.41–196.03	0.165
LINC00608	0.04	0–0.74	0.03	0.01	0–0.27	0.006^**^
LINC01300	9.26	1.67–51.37	0.011	39.02	3.79–401.34	0.002^**^
MIR99AHG	0.19	0.05–0.69	0.012	0.23	0.05–0.97	0.046^*^

* P < 0.05,

** P < 0.01,

*** *P < 0.001*.

### Development of the Gene Signature Prediction Model

The overall score of these 8 genes based on regression coefficients was as follows: signature score = (−0.9250632 × expression of AC008655.1) – (4.7457363 × expression of AC010624.1) – (4.1420857 × expression of AC055758.2) – (4.1541207 × expression of AC118553.1) + (1.8755252 × expression of AC130456.4) – (3.5253117 × expression of LINC00608) + (3.3913564 × expression of LINC01300) – (1.8634876 × expression of MIR99AHG).

**Figure 3:**
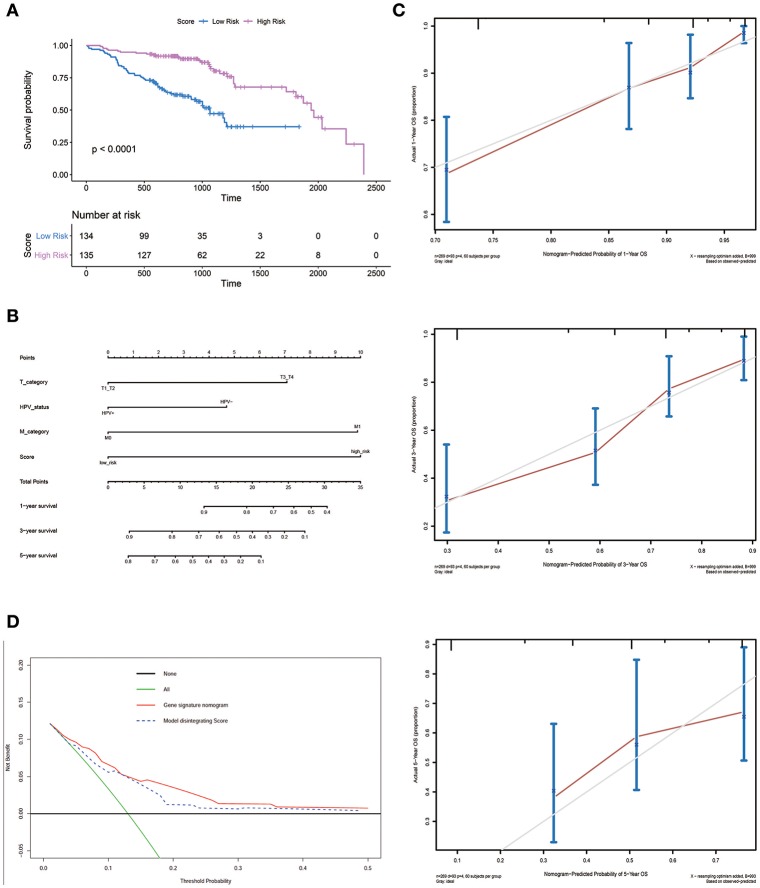
Nomogram plot for patients with Head and Neck Squamous Cell Carcinoma. **(A)** Overall survival analysis on signature score in the GSE65858 dataset. **(B)** The gene nomogram was developed with signature score, T category, M category and HPV status. **(C)** Calibration curves of the gene nomogram. The y-axis represents the actual overall survival rate. The x-axis represents the predicted overall survival rate. The gray diagonal represents a perfect prediction of an ideal model. Dark red solid lines indicate the performance of the nomogram, where closer to the diagonal dashed line indicates a better prediction. **(D)** Decision curve analysis for the gene signature nomogram and the model without Score. The y-axis measures the net benefit. The red solid line represents the gene signature nomogram. The blue dashed line represents model without Score. The green line represents the assumption that all patients have died. Thin black line represents the assumption that no patients have died.

An optimal cutoff value was selected to separate patients into low-risk and high-risk groups using the pROC package in R. Figure 3A shows that patients in the low-risk group had longer OS (*p* < 0.0001) than those of the high-risk group. Multivariate cox regression analysis included Score, Uicc_stage, T_category, N_category, M_category, and HPV_status as independent predictors. The results showed that factors with *P* < 0.05 were included in the prediction model (Table 3). Then, the model was also presented as a nomogram (Figure 3B). Figure 3C shows that calibration of the new prediction model was fitted; the results predicted by the model were basically consistent with the ideal results of the model incorporating the gene signature (C-index 0.75, 95% CI 0.73–0.78), which was more predictive than models not including it (C-index 0.68, 95% CI 0.65–0.71; Table 3).

**Table 3:** Model discussion for 8 lncRNA HNSCC.

**Characteristics**	**Model 1**			**Model 2**		
	**Hazard Ratio**	**95% CI**	***P*-value**	**Hazard ratio**	**95% CI**	***P*-value**
HPV_status	0.53	0.31–0.9	0.018^*^	0.53	0.31–0.9	0.018^*^
M_category	3.29	1.41–7.63	0.006^**^	3.47	1.5–8.01	0.004^**^
N_category	1.18	0.66–2.1	0.576	1.27	0.71–2.26	0.425
T_category	2.42	1.28–4.58	0.007^**^	2.41	1.27–4.57	0.007^**^
Uicc_stage	1.03	0.37–2.88	0.949	0.92	0.33–2.57	0.874
Score				0.28	0.17–0.45	0^***^
C-index	0.68 (0.65–0.70)			0.75 (0.73–0.78)		

* P < 0.05,

** P < 0.01,

*** *P < 0.001*.

### Clinical Use

Figure 3D shows the DCAs for the prognostic prediction model and the model without the gene signature. The results showed that using the prognostic prediction model with the gene signature to predict the OS of patients could be of more benefit in the current model than the treat-all patients- or treat-none scheme. Compared with this model without the gene signature, the prognostic prediction model with the gene signature could bring greater benefits to patients.

### GSEA of the Signature Score

**Figure 4:**
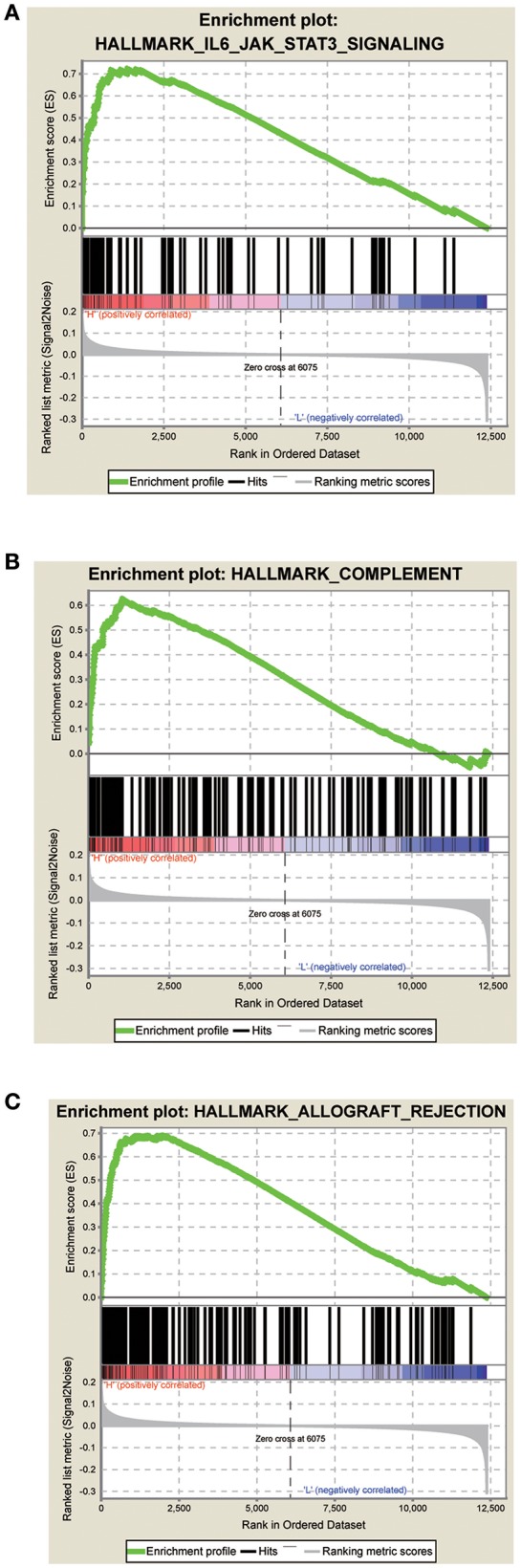
Score correlated enrichment gene analysis with GSEA. **(A)** Hallmark IL6 JAK SATA3 SIGNALING (*P* = 0.004; FDR = 0.104; ES = 0.72); **(B)** HALLMARK COMPLEMENT (*P* = 0.002; FDR = 0.110; ES = 0.62); **(C)** HALLMARK ALLOGRAFT REJECTION (*P* = 0.031; FDR = 0.180; ES = 0.69).

The GSEA data showed that samples with high signature scores were mainly enriched in the hallmark of IL6 JAK SATA3 signaling, HALLMARK COMPLEMENT, and HALLMARK ALLOGRAFT REJECTION (*P* < 0.05; FDR < 0.2; Figure 4; Table 4).

**Table 4:** Gene Set Enrichment Analysis (GSEA) group by score.

**GS follow link to MSigDB**	**SIZE**	**ES**	**NES**	**NOM *p*-val**	**FDR *q*-val**	**FWER *p*-val**	**Rank at max**	**Leading edge**
HALLMARK_IL6_JAK_STAT3_SIGNALING	66	0.72197646	1.6556728	0.004040404	0.104168214	0.065	1,383	Tags = 42%, list = 11%, signal = 48%
HALLMARK_COMPLEMENT	144	0.6246483	1.593254	0.002061856	0.1100425	0.138	1,045	Tags = 34%, list = 8%, signal = 37%
HALLMARK_ALLOGRAFT_REJECTION	146	0.6888915	1.5099503	0.031189084	0.18018493	0.287	1,987	Tags = 51%, list = 16%, signal = 60%

### Weighted Co-expression Network Construction (WGCNA)

**Figure 5:**
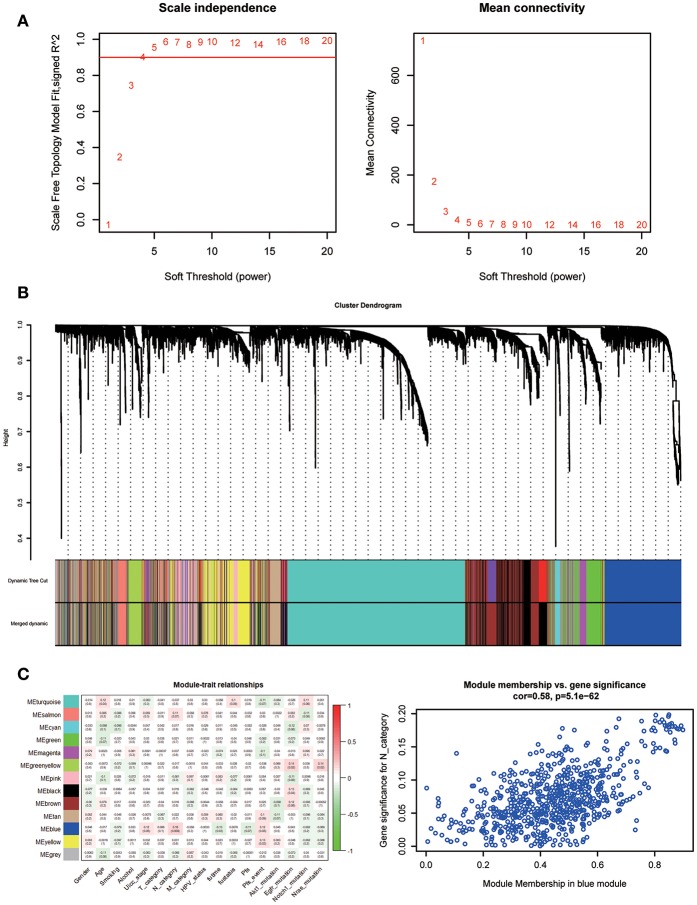
Determination of soft-thresholding power in WGCNA. **(A)** Analysis of the scale-free fit index for various soft-thresholding powers (β) and analysis of the mean connectivity for various soft-thresholding powers. **(B)** Dendrogram of all differentially expressed genes clustered based on a dissimilarity measure. **(C)** Heatmap of the correlation between module eigengenes and clinical traits of HNSC. Scatter plot for correlation between gene module membership in the blue module and gene significance.

A co-expression network was constructed using GSE65858, including 269 HNSCC samples with complete clinical data. The expression amounts of 5,000 genes including 4,992 mRNAs and 8 lncRNAs were analyzed for co-expression network constructing the “WGCNA” package. In the current study, to ensure a scale-free network, we selected β = 5 as the soft-thresholding power (Figure 5A) and identified a total of 13 modules (Figure 5B).

### Identification of Hub lncRNAs in Modules and Function Annotation

Identifying modules most significantly related to clinical features is difficult. We found that the blue module was correlated with N category, T category, and Stage; N category showed the highest correlation (*P* = 5.1e-62; *r* = 0.58, Figure 5C).

**Figure 6:**
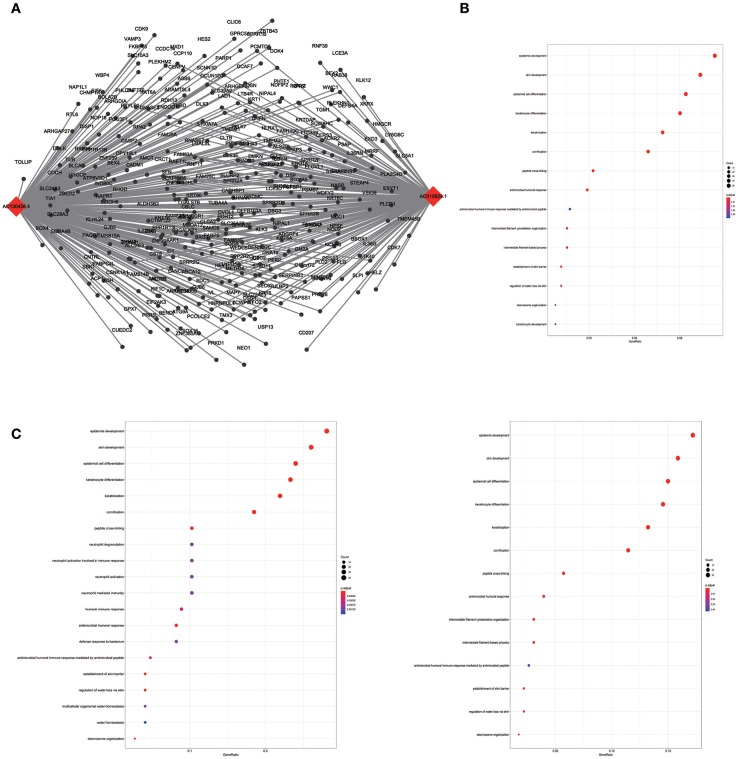
LncRNA-mRNA network in Black module and functional enrichment. **(A)** The lncRNA-mRNA network in Black module, **(B)** GO-BP enrichment analysis (All genes in Black module), **(C)** GO-BP enrichment analysis (AC01 0624.1 related genes in Black module), and **(D)** GO-BP enrichment analysis (AC130456.4 related genes in Black module).

**Figure 7:**
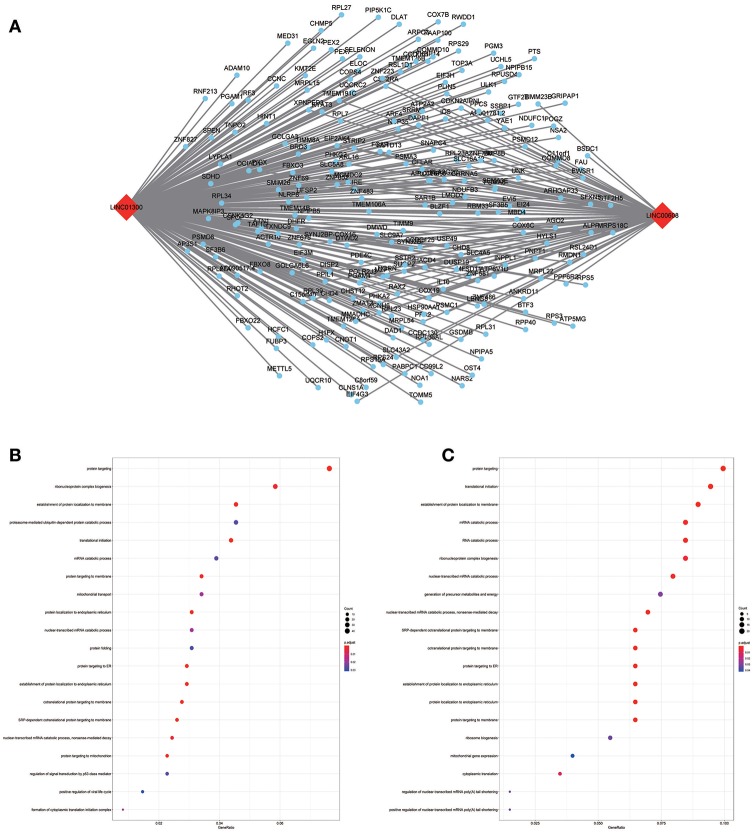
LncRNA-mRNA network in Blue module and functional enrichment. **(A)** The lncRNA-mRNA network in Blue module, **(B)** GO-BP enrichment analysis (All genes in Blue module), **(C)** GO-BP enrichment analysis (LINC01300 related genes in Blue module).

**Figure 8:**
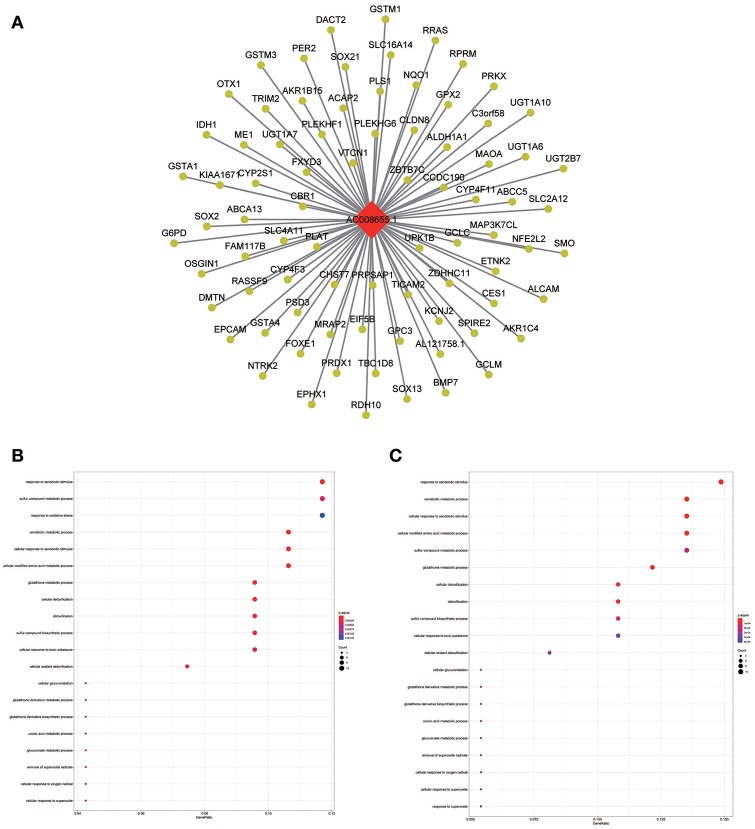
LncRNA-mRNA network in Tan module and functional enrichment. **(A)** The lncRNA-mRNA network in Tan module, **(B)** GO-BP enrichment analysis (All genes in Tan module), and **(C)** GO-BP enrichment analysis (AC008655.1 related genes in Tan module).

**Figure 9:**
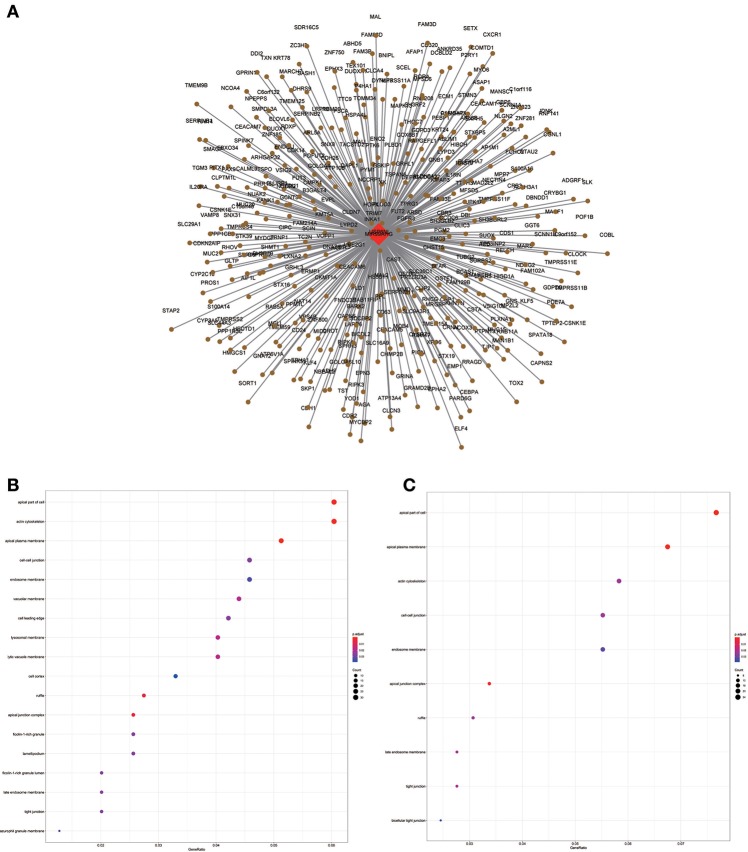
LncRNA-mRNA network in Brown module and functional enrichment. **(A)** The lncRNA-mRNA network in Brown module, **(B)** GO-CC enrichment analysis (All genes in Brown module), and **(C)** GO-CC enrichment analysis (MIR99AHG related genes in Brown module).

**Figure 10:**
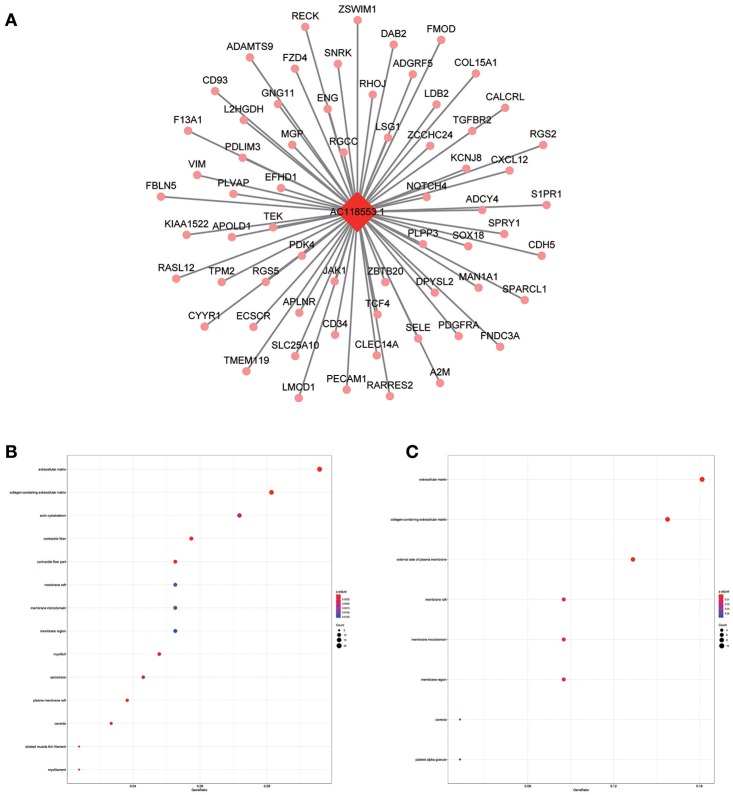
LncRNA-mRNA network in Magenta module and functional enrichment. **(A)** The lncRNA-mRNA network in Magenta module, **(B)** GO-CC enrichment analysis (All genes in Magenta module), and **(C)** GO-CC enrichment analysis (AC118553.1 related genes in Magenta module).

To assess the functional involvement of the hub lncRNAs, their co-expression modules were determined via the WGCNA algorithm. We found AC010624.1 and AC130456.4 in the black module, LINC00608 and LINC01300 in the blue module, MIR99AHG in the brown module, AC008655.1 in the tan module, and AC118553.1 in the magenta module. All mRNAs in the modules, as well as mRNAs associated with the hub lncRNAs assessed by the ClusterProfiler Package in the R software, are shown in Figures 6–10.

### Validation of Hub lncRNAs

**Figure 11:**
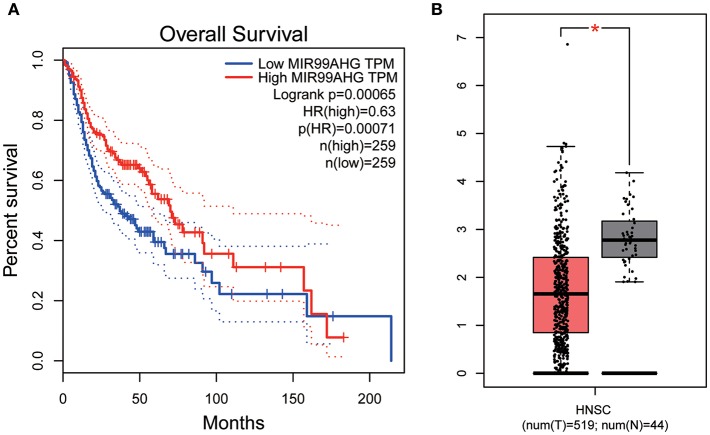
Overall survival analyses and Gene expression difference analysis on MIR99AHG in the TCGA data set. **(A)** Kaplan-Meier curves of overall survival according to expression of MIR99AHG in TCGA-HNSC database. **(B)** Differentially expressed MIR99AHG in HNSC and normal tissues (*P* < 0.05).

All hub lncRNAs were selected for validation using the TCGA and GTEx datasets. In the TCGA database, MIR99AHG expression was associated with overall survival (Figure 11A), with a difference in expression between cancer and adjacent tissues (Figure 11B).

## Discussion

Using the mRNAs and lncRNAs selected after re-annotating the GSE65858 dataset of the GEO database, 8 prognosis-related lncRNAs including AC010624.1, AC130456.4, LINC00608, LINC01300, MIR99AHG, AC008655.1, AC055758.2, and AC118553.1 were obtained by univariate analysis, cox LASSO regression and multivariate analysis. Combined with clinical information, a nomogram containing an 8-lncRNA signature was established. Time-dependent calibration curves and decision curve analysis confirmed the prognostic significance and prediction superiority of the signature and nomogram. Further, GSEA of the signature score indicated that samples with high scores were mainly enriched in IL6/JAK/SATA3 signaling, complement, and allograft rejection, indicating HNSCC patients with poor prognosis might have dysfunctional immune systems. To identify the potential functions and involved biological processes of each lncRNA in the signature, WGCNA was performed. The black module containing AC010624.1 and AC130456.4, the blue comprising LINC00608 and LINC01300, the brown containing MIR99AHG, the tan encompassing AC008655.1, and the magenta containing AC118553.1 were obtained. GO enrichment analysis of all genes and lncRNA associated genes in modules was performed. Compared with previously established signatures (20–23), the current signature was more effective in predicting the prognosis of patients with HNSCC; more importantly, it indicated that the poor prognosis of high-score patients may be associated with changes in immune function. Furthermore, the possible biological functions of the lncRNAs contained in the signature were assessed, which provides new insights into possible treatment directions for HNSCC patients.

Previous studies have shown that the IL6/JAK/SATA3 pathway plays an important role in HNSCC (25–27), corroborating the current GSEA data. Drugs targeting the IL6/JAK/SATA3 pathway with low side effects are still under investigation (25–27). It can be inferred from the above signature that patients with high scores may benefit more from targeted drugs against the IL6/JAK/SATA3 pathway, which has a high guiding significance in future clinical applications. In addition, immune cells in the microenvironment such as M1/M2 macrophage, CD8+ T lymphocyte, NK cells, etc. can affect the tumor growth, progression, and cachexia of HNSCC by secreting IL-6. M2 macrophage can promote the proliferation of HNSCC, while CD8+ T lymphocyte, M1 macrophage, and NK cells can inhibit the progression of HNSCC via IL-6 (28). Therefore, we predicted the immune-related cells in the microenvironment of HNSCC through CIBERSORT and explored the relationship between lncRNAs in signature and these immune cells. It was found that lncRNA associated with good prognosis of HNSCC, such as AC010624.1 and MIR99AHG, was negatively correlated with NK cells resting and M2 macrophage (Figures S1A–D). Similarly, LINC01300 related to poor prognosis of HNSCC was negatively correlated with CD8+ T lymphocyte and M1 macrophage (Figures S1E,F). It provides a basis for the functional study of these lncRNAs in the IL6/JAK/STAT3 pathway and immune microenvironment, but the mechanism among lncRNAs and IL-6 remains to be explored. Besides, it had been reported HNSCC patients with high tumor mutational burden (TMB) appears worse prognosis (29). Our results showed AC010624.1 was negatively related with TMB, while LINC01300 was positively related with TMB (Figures S1G,H). LncRNAs in the signature play an important role in maintaining immune function in HNSCC, and the mechanisms need to be explored in the future.

WGCNA results showed that the black module containing AC010624.1 and AC130456.4 was closely related to EGFR mutation in clinical phenotypes, while GO enrichment data indicated that these two lncRNAs may both participate in epidermal cell differentiation and skin development, which is consistent with the potential function of the black module. EGFR is elevated in ~90% of HNSCC patients and considered a negative prognostic factor for patients with HNSCC (30–32). Combining these findings, we hypothesized that AC010624.1 and AC130456.4 affect prognosis by participating in epidermal cell differentiation and skin development, which in turn affects EGFR mutation in patients with HNSCC. Whether and how AC010624.1 and AC130456.4 affect EGFR mutations deserves further investigation by molecular biology experiments.

Similarly, the brown module containing MIR99AHG was closely related to EGFR mutation in clinical phenotype. GO enrichment results indicated that the brown module was involved in the formation of cellular structures, such as the apical part of the cell, actin cytoskeleton, and apical plasma membrane, and MIR99AHG might also participate in this biological function. Previous studies have reported that MIR99AHG, also known as MONK, is a good prognostic indicator of breast cancer, lung squamous cell carcinoma, and colorectal cancer (33–36). Our results also supported these conclusions. We further investigated the potential biological process by which MIR99AHG acts and may affect EGFR gene mutation. There is a great need to further explore the role of MIR99AHG in the prognosis evaluation and treatment of HNSCC and other cancers. Finally, we used the TCGA database for validation. Patients with high MIR99AHG expression had better OS, and MIR99AHG was differentially expressed between HNSCC and adjacent tissues. In the future, the biological significance of MIR99AHG in HNSCC should be assessed.

The blue module containing LINC00608 and LINC01300 was associated with T and N stages, and GO enrichment results showed the blue module may be involved in protein processing and presentation. The potential functions of both LINC00608 and LINC01300 were consistent with the blue module. The potential function enriched for AC008655.1 was the same as which the tan module, both of which might be related to the response to xenobiotics. The magenta module and AC118553.1 may be involved in the regulation of the extracellular matrix. LINC01300, AC008655.1, and AC118553.1 have not been previously reported. For the first time, we predicted the biological processes that may involve these lncRNAs, which deserve further investigation for predicting the prognosis of patients with HNSCC.

However, the limitations of this study should be mentioned. First, we did not apply an additional data set to validate the novel signature, which needs to be validated in large cohorts. Secondly, alcohol and tobacco consumptions are known prognostic factors of HNSCC (37–39), but the present signature did not fully consider the impact of these factors. This should be investigated in the future. Thirdly, HNSCC originated from different tissues, but the dataset we applied does not contain this information. Our signature lacked information of origination. Finally, we did not perform molecular biology experiments and clinical specimen validation to further explore the biological functions of hub lncRNAs involved in HNSCC.

In conclusion, we established an 8-lncRNA signature and a nomogram for predicting the prognosis of patients with HNSCC, and speculated that patients with a high signature score may have dysfunctional immune regulation, which may be a new direction of treatment for patients with HNSCC. Functional enrichment was performed to predict the potential functions of the lncRNAs contained in the signature, and for the first time, various biological functions and processes potentially altered by multiple lncRNAs were revealed. The 8-lncRNA signature is of great significance in evaluating patient prognosis and exploring new therapeutic targets for patients with HNSCC.

## Data Availability

Publicly available datasets were analyzed in this study. This data can be found here: https://www.ncbi.nlm.nih.gov/geo/query/acc.cgi?acc=GSE65858.

## Author Contributions

ZL conceived the study. BY conducted all statistical analyses. JS reviewed relevant literature and drafted the manuscript. LX, YC, XC, XQ, YL, and YT provided guidance to the study. All authors read and approved the final manuscript.

### Conflict of Interest Statement

The authors declare that the research was conducted in the absence of any commercial or financial relationships that could be construed as a potential conflict of interest.

## Supplementary Material

Figure S1 Relationship between lncRNA AC010624.1 and **(A)** CD8+ T lymphocyte, **(B)** M2 macrophage. Relationship between lncRNA MIR99AHG, and **(C)** CD8+ T lymphocyte, **(D)** NK cells resting. Relationship between lncRNA LINC01300 and **(E)** CD8+ T lymphocyte, **(F)** M1 macrophage. Relationship between lncRNA **(G)** AC010624.1, **(H)** LINC01300, **(I)** MIR99AHG and tumor mutational burden (TMB).

Table S1 The basic characteristics of the patients.
