# Supplementary figures and images for: Genome-Wide Identification of a Novel Eight-lncRNA Signature to Improve Prognostic Prediction in Head and Neck Squamous Cell Carcinoma
Source: Front Oncol. 2019 Sep 18;9:898. doi: 10.3389/fonc.2019.00898 (PMC6759597; doi:10.3389/fonc.2019.00898)

(A)

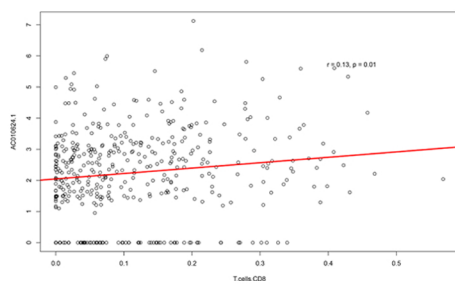

(B)

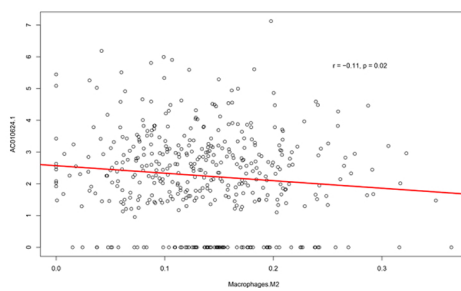

(C)

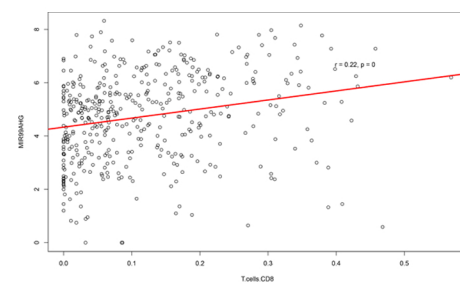

(D)

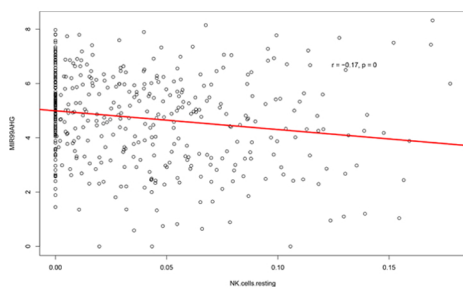

(E)

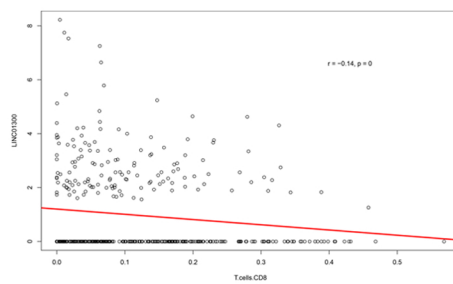

(F)

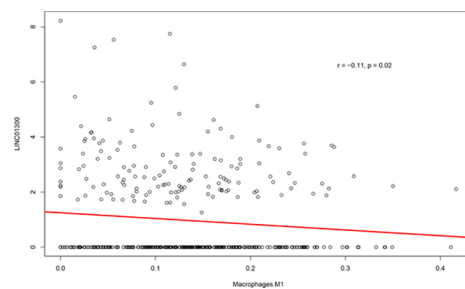

(G)

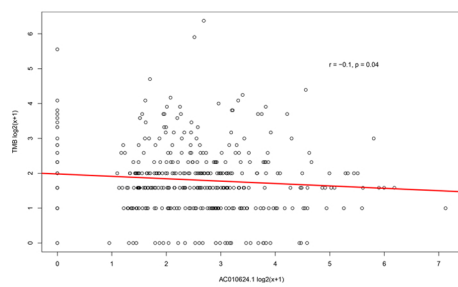

(H)

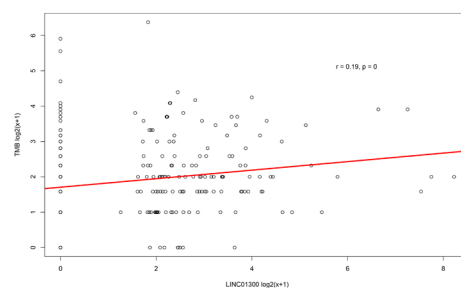

(I)

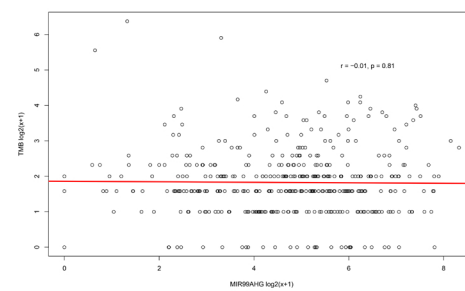

Supplement: Figure S1 — Relationship between lncRNA AC010624.1 and (A) CD8+ T lymphocyte, (B) M2 macrophage. Relationship between lncRNA MIR99AHG, and (C) CD8+ T lymphocyte, (D) NK cells resting. Relationship between lncRNA LINC01300 and (E) CD8+ T lymphocyte, (F) M1 macrophage. Relationship between lncRNA (G) AC010624.1, (H) LINC01300, (I) MIR99AHG and tumor mutational burden (TMB). [file Data_Sheet_1.PDF]
